# Physical, functional and conditional interactions between ArcAB and phage shock proteins upon secretin-induced stress in *Escherichia coli*

**DOI:** 10.1111/j.1365-2958.2009.06809.x

**Published:** 2009-08-04

**Authors:** Goran Jovanovic, Christoph Engl, Martin Buck

**Affiliations:** Division of Biology, Sir Alexander Fleming Building, Imperial College LondonSouth Kensington Campus, London SW7 2AZ, UK.

## Abstract

The phage shock protein (Psp) system found in enterobacteria is induced in response to impaired inner membrane integrity (where the Psp response is thought to help maintain the proton motive force of the cell) and is implicated in the virulence of pathogens such as *Yersinia* and *Salmonella*. We provided evidence that the two-component ArcAB system was involved in induction of the Psp response in *Escherichia coli* and now report that role of ArcAB is conditional. ArcAB, predominantly through the action of ArcA regulated genes, but also via a direct ArcB–Psp interaction, is required to propagate the protein IV (pIV)-dependent *psp*-inducing signal(s) during microaerobiosis, but not during aerobiosis or anaerobiosis. We show that ArcB directly interacts with the PspB, possibly by means of the PspB leucine zipper motif, thereby allowing cross-communication between the two systems. In addition we demonstrate that the pIV-dependent induction of *psp* expression in anaerobiosis is independent of PspBC, establishing that PspA and PspF can function as a minimal Psp system responsive to inner membrane stress.

## Introduction

Bacteria in their native habitats encounter a plethora of stresses, which challenge their cellular integrity. Robustness of the cytoplasmic membrane is required for it to function as a barrier between the inside of the cell and the environment. Bacteria have therefore evolved a number of systems to maintain membrane integrity, one of which is the phage shock protein (Psp) response, highly conserved in Gram-negative bacteria. In *Escherichia coli*, Psp genes are organized in a regulon (*pspF pspABCDE* and *pspG*) that is under the control of a sigma54-dependent promoter activated by PspF, positively controlled by PspBC and negatively regulated by PspA (reviewed by Model *et al*., 1997; Darwin, 2005; Wigneshweraraj *et al*., 2008). Psp expression is induced upon inner membrane (IM) stress which dissipates the proton motive force (pmf) (potentially resulting in a reduced energy status of the cell). Psp effector proteins (e.g. PspA – effector upon stress, PspD and PspG) subsequently help to conserve pmf (Kleerebezem *et al*., 1996; Jovanovic *et al*., 2006; Kobayashi *et al*., 2007). Understanding the mechanism of Psp induction and its biological function is important since the Psp system is significant in protein translocation and in the growth and virulence of pathogenic enterobacteria (reviewed by Darwin, 2005; 2007; Rowley *et al*., 2006). The PspA homologue Vipp1 has been shown to be essential in plants and *Synechocystis* for thylakoid biogenesis and hence photosynthesis (Westphal *et al*., 2001). Further, PspA homologues have been reported in Gram-positive bacteria and archea (Bidle *et al*., 2008; Vrancken *et al*., 2008). PspA therefore appears to have a fundamental role in the three domains of life.

**Fig. 2:**
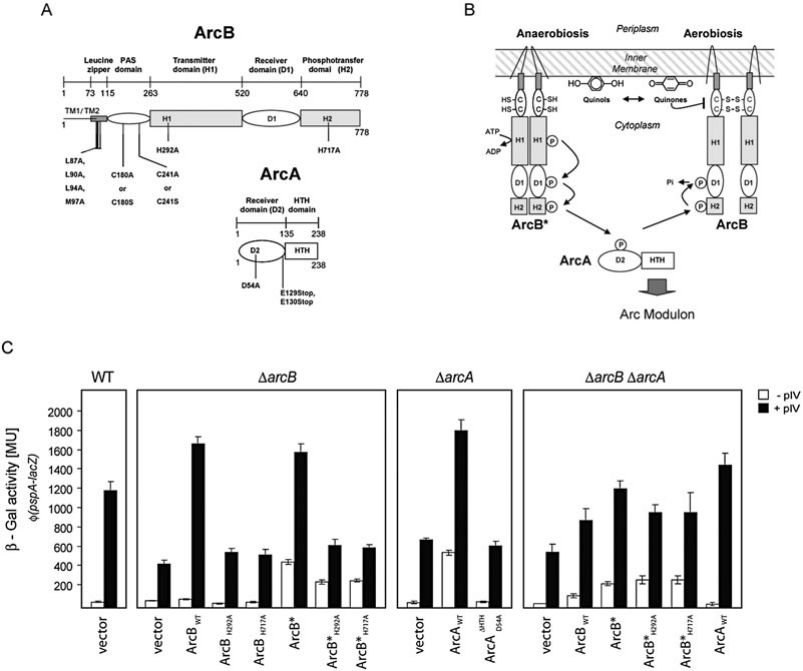
Activation of ArcAB is required for robustness of pIV-dependent induction of *psp* expression. A. Schematic representation of ArcB and ArcA (adapted from Malpica *et al*., 2004) with the locations of the amino acid substitutions used in this study. ArcB: activation mutants – C180A/S and C241A/S substitutions in PAS domain; phosphorelay mutants – H292A or H717A; leucine zipper mutant (LeuZm) – L87A, L90A, L94A and M97A. ArcA: a phosphorylation-defective mutant – D54A, and DNA-binding-deficient mutant (introduction of two stop codons, E129Stop and E130Stop) –ΔHTH. B. Regulation of the ArcAB system. ArcB is an IM-localized dimer. Its kinase activity is diminished by UQ and elevated in the presence of UQH_2_ leading to reduction of the ArcB Cys180 and Cys241 thiol groups (in this article functionally equivalent to ArcB* form) in PAS (Per-Arnt-Sim) domain and an increased interaction between the two ArcB TM domains. (PAS domains are signalling domains and they function as input modules in proteins that sense oxygen, redox potential, light and several other stimuli.) Reduction of ArcB is followed by autophosphorylation of H292 (H1) and phosphorelay via D576 (D1) and H717 (H2) to residue D54 (D2) within the response regulator ArcA. C. Bipartite role of ArcB activation in pIV-dependent induction of *psp* expression. Induction of *psp* expression in Δ*arcB* (MVA63), Δ*arcA* (MVA79) and a double mutant Δ*arcAB* (MVA94) strains expressing ArcB_WT_, ArcA_WT_ and their mutants was measured using β-Gal assays under microaerobic conditions in the absence or presence of pIV. WT – wild-type strain (MVA44); vector – pCA24N.

The nature of the stress signal and how it is detected by the Psp system remains unknown (reviewed by Darwin, 2005). Previously we observed that ArcB (the sensor kinase of the two-component ArcAB system) is required for full protein IV (pIV)-dependent *psp* expression in *E. coli* and proposed that *psp*-inducing stresses that dissipate pmf may activate ArcB (Jovanovic *et al*., 2006). The ArcAB system regulates the transition from aerobic to anaerobic respiration and fermentation (reviewed by Malpica *et al*., 2006) where ArcB has been shown to sense the redox state of the cell through changes in the ubiquinone : ubiquinol (UQ : UQH_2_) ratio (see Fig. 2A and B) (Georgellis *et al*., 2001; Malpica *et al*., 2004). Increased levels of UQH_2_ attenuate inhibition on ArcB kinase activity imposed by UQ, thus enabling ArcB to phosphorylate ArcA (the response regulator). Phosphorylated ArcA (ArcA-P) then represses genes involved in aerobic respiration and upregulates genes involved in anaerobic respiration and fermentation. Notably, the ArcAB system is also known to be of particular importance under microaerobic growth conditions, where oxygen is limiting rather than being completely absent (Alexeeva *et al*., 2003).

Recently, ArcB-independent *psp* induction was reported (Seo *et al*., 2007). We therefore explored further the relationship(s) between the Psp and Arc system under different growth conditions and now report that involvement of Arc is conditional, consistent with both observations (Jovanovic *et al*., 2006; Seo *et al*., 2007). We find that ArcAB is required to propagate pIV-dependent induction of the Psp response under microaerobic, but not under aerobic or anaerobic growth conditions. The Arc system appears to be important for Psp-signal amplification, via the ArcB–ArcA phosphorelay. In addition, activated ArcB is by itself observed to elevate *psp* expression. Further, we have identified a direct binding interaction between ArcB and PspB that supports part of a Psp-specific signal transduction pathway used under microaerobic growth conditions. Notably in anaerobiosis, pIV-dependent *psp* expression is largely independent of PspBC (two positive regulators of the Psp response) and ArcAB, suggesting either that the repressive PspA–PspF complex is itself capable of recognizing inducing signals, or that PspBC and ArcAB are substituted by other gene products.

## Results

### PIV-dependent induction of *psp* expression under different growth conditions

**Fig. 1:**
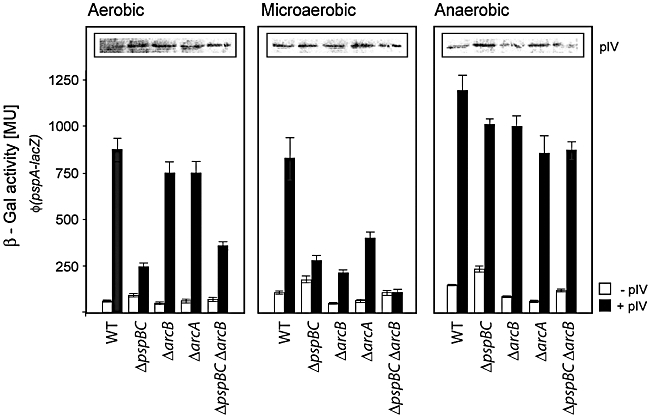
pIV-dependent induction of *psp* expression under different growth conditions. pIV secretin (from pGJ4) induced *psp* expression in *E. coli*[MG1655 φ(*pspA–lacZ*); MVA44] cells grown under aerobic, microaerobic or anaerobic conditions as measured using a β-Gal assay (see *Experimental procedures*). WT – wild type, MVA44. Insets: Western blot analyses of pIV expression in corresponding cells using antibodies against pIV (α-pIV) (see *Experimental procedures*). The relative expression levels (in arbitrary units) of pIV were determined as follows: aerobic – 1.57, 2.00, 1.93, 1.84, 2.03; microaerobic – 2.13, 2.27, 2.17, 2.32, 1.77; anaerobic – 1.71, 2.35, 1.82, 2.25, 1.77, and illustrate that the overall variability in pIV expression is ∼1.5-fold.

We measured pIV-dependent *psp* induction in strains with different complements of *arc* and *psp* genes grown under aerobic, microaerobic and anaerobic conditions (see *Experimental procedures*) where the ArcAB proteins are known to have differing importance (Fig. 1). We found that *psp* induction is dependent on ArcAB only in microaerobiosis and on PspBC (positive regulators) in microaerobiosis and aerobiosis (Fig. 1). In aerobiosis, a requirement for the ArcAB system is not apparent (Fig. 1). Under anaerobic growth, full pIV-dependent *psp* induction is largely independent of PspBC and the Arc system, when compared with microaerobiosis (Fig. 1). These differing dependencies on Arc are consistent with the ArcAB system functioning as a microaerobic redox regulator, required under microaerobic but not aerobic or anaerobic conditions (Alexeeva *et al*., 2003). Notably, under microaerobic growth conditions, the requirement for ArcB is more pronounced then for ArcA. Importantly, control reactions demonstrate that the different growth conditions *per se* do not affect the activity from the *psp* promoter since *psp* expression in Δ*pspA* cells is similar (Fig. S1A). Further since the level of *psp* expression in control strains Δ*pspA*Δ*arcB*φ(*pspA–lacZ*) and Δ*pspA*Δ*arcA*φ(*pspA–lacZ*) are unaffected by ArcB and ArcA, we infer that the Arc proteins may contribute to relieving PspA-negative regulation in microaerobiosis (Fig. S1A). In *psp* induction assays we used the outer membrane (OM) secretin, pIV, to induce *pspA–lacZ* expression. It is believed that pIV mislocalizes to the IM thereby impairing cell membrane integrity and resulting in a *psp*-inducing stress signal similar to that caused by PulD (Guilvout *et al*., 2006). To ascertain that the strains assayed were exposed to equivalent (pIV-induced) levels of ‘stress’, we tested pIV expression and determined the amount of pIV localized within the IM. As shown in Fig. 1, pIV expression did not change greatly in the different backgrounds tested or when the cells were grown under differing aeration. Additionally, the distribution of pIV between the IM and OM was similar in microaerobiosis in the wild-type (WT), *arcA*, *arcB* and *pspBC* deletion backgrounds (Fig. S1B).

Since Psp effector proteins (PspA, PspD and PspG) alter expression levels of genes associated with aerobic growth (Jovanovic *et al*., 2006) and ArcAB regulates the switch from aerobic to anaerobic respiration and fermentation, we tested whether (i) pIV changes oxygen consumption in microaerobiosis (Fig. S1C) and (ii) other global regulators associated with aerobicity (e.g. FNR or Nar; see Fig. S1D) also affect *psp* induction. The results show that pIV changes oxygen consumption in an ArcB-dependent manner and that among the global regulators, the Arc system has the greatest effect on *psp* expression, supporting the idea of specific cross-talk between the Psp and ArcAB systems. Taken together, these results indicate a growth condition-specific requirement for ArcAB and PspBC proteins in *psp* induction. In addition, since under anaerobiosis induction of *psp* is independent of the PspBC-positive regulators we suggest that the PspA–F regulatory complex may itself be capable of perceiving a pIV-dependent inducing signal. Importantly, all subsequent experiments reported here were conducted under the microaerobic conditions where the roles of PspBC and ArcAB were most prominent.

### Activation of ArcA facilitates pIV-dependent induction of *psp* expression

To further assess the roles of ArcAB in pIV-dependent induction of *psp* expression in microaerobiosis, we constructed mutants of ArcB (see Table 1) and ArcA that: (i) had a constitutively active kinase: ArcB_C180A/C241A_ (termed ArcB*), (ii) disrupted the phosphorelay from ArcB to ArcA: ArcB_H292A_, ArcB_H717A_, ArcB*_H292A_ and ArcB*_H717A_, and (iii) inactivated ArcA (by disrupting phosphorylation and preventing binding to target promoters): 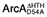 (Fig. 2A). Initially we determined that WT and mutant forms of ArcB proteins were similarly expressed (Fig. S2Ai and data not shown) and appropriately localized within the IM (Fig. S2Aii and data not shown). As a further measure of functionality, we showed that the WT and certain mutant ArcB and ArcA proteins (only those with intact phosphorelay systems) were able to activate ArcA-P-dependent *pfl* transcription (Fig. S2B). We then assayed the ability of Arc mutants to complement Δ*arcB* or Δ*arcA* mutations and support pIV-dependent induction of *psp* expression. As shown in Fig. 2C, ArcB_WT_ and ArcB* or ArcA_WT_ fully complemented Δ*arcB* or Δ*arcA* mutations, respectively, for pIV-dependent induction of *psp* expression. None of the phosphorelay mutants in ArcB or the inactive ArcA variant fully supported pIV-dependent induction of *psp* expression (Fig. 2C), indicating that ArcA-P has a role in promoting pIV-dependent induction. Importantly, control reactions demonstrated that similar levels of pIV secretin were present in all the samples tested (Fig. S2C and data not shown). Overexpression of ArcB proteins in a Δ*arcAB* strain showed that in the absence of ArcA_WT_, the ArcB_WT_ and ArcB* similarly to ArcB* phosphorelay mutants cannot fully support pIV-dependent induction of *psp* expression (compared with results obtained with a Δ*arcB*) and that ArcB* and its phosphorelay mutants promote an ArcA- and pIV-independent low-level induction of *psp* (Fig. 2C). These data indicate that ArcB has an activity with respect to Psp that is partially ArcA-independent. In contrast, overexpression of ArcA_WT_ in the Δ*arcAB* strain led to strong pIV-dependent induction of *psp* (Fig. 2C), indicating that there are two levels of Arc involvement in *psp* expression, one mediated by ArcB independent of ArcA, and one directed by ArcA.

**Table 1:** Biochemical phenotypes of *arcB* alleles.

Protein	Motif(s) mutation	ArcB activation^a^	ArcB constitutively active^b^	ArcA activation^c^
ArcB_WT_	Wild type	+	−	+
ArcB_H292A_	H292A	+	−	−
ArcB_H717A_	H717A	+	−	−
ArcB*	C180A C241A	−	+	+
ArcB*_H292A_	C180A C241A H292A	−	+	−
ArcB*_H717A_	C180A C241A H717A	−	+	−
ArcB^LeuZm^	L87A L90A L94A M97A	+	+	+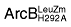	L87A L90A L94A M97A H292A	+	+	−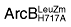	L87A L90A L94A M97A H717A	+	+	−

a Kinase ‘on’, wild-type C180 and C241 (apo form).

b Kinase constitutively ‘on’.

c Phosphorelay ‘on’.

### An active form of ArcB promotes low-level induction of *psp* expression independent of pIV

In the absence of pIV (white bars in Fig. 2C) we observed that overexpression of either ArcB_WT_ or the ArcB phosphorelay mutants (ArcB_H292A_ or ArcB_H717A_) did not significantly induce *psp* expression. However, overexpression of either activated ArcB (ArcB*) or the ArcB* phosphorelay mutants (e.g. ArcB*_H717A_) were able to induce a low level of *psp* expression (compared with ArcB_WT_). When ArcB* is overexpressed in the Δ*arcAB* strain, a similar low level of pIV-independent *psp* expression was observed (Fig. 2C). We reasoned that the low-level induction of *psp* expression caused by the ArcB* phosphorelay mutants appears to be due to an effect of activated ArcB*, which is completely independent of ArcA or ArcA-P action.

Overexpression of ArcA_WT_ in a Δ*arcA* strain in the absence of pIV results in induction of *psp* expression (Fig. 2C). Notably, this elevation of *psp* expression by overexpression of ArcA_WT_ is ArcB-dependent (compare Δ*arcA* and Δ*arcAB* strains) (Fig. 2C). However in the presence of pIV, overexpression of ArcA_WT_ in the Δ*arcAB* strain results in strong *psp* expression even in the absence of ArcB (although we note that this activity could be due to ArcA-P formation arising from cross-talk or acetyl phosphate). Together with the results implying that low-level induction of *psp* expression can occur in an ArcA-independent manner in the presence of the ArcB* phosphorelay mutants, these data again strongly suggest that activation of the Arc system results in two distinct outcomes (one via ArcA-P, the other via an active form of ArcB) that generate the Arc-dependences in the signalling pathway for pIV-dependent induction of *psp* expression.

### Activation of ArcAB results in decreased electron potential (Δψ)

**Fig. 3:**
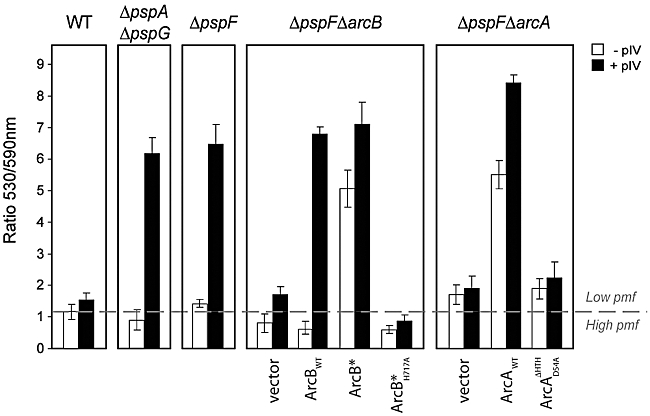
Activation of ArcAB reduces the electron potential (Δ*ψ*). Cells were grown microaerobically, treated with JC-1 dye, and the change in Δ*ψ* calculated as the 530/590 nm (green/red) ratio. ArcB_WT_, ArcA_WT_ and key variants were expressed in the absence or presence of pIV. An increase in the 530/590 ratio indicates a decrease in Δ*ψ* compared with WT (*E. coli* MG1655) with vector plasmid pCA24N.

To address the nature of the signal that is responsible for low-level induction of *psp* expression by ArcB* and to potentially discriminate between roles of ArcB* and ArcA-P, we measured the electron potential (Δ*ψ*) of strains expressing either WT or mutant ArcB or ArcA proteins in the presence and absence of pIV (Fig. 3).

In the absence of a functioning Psp system (i.e. no effectors present, Δ*pspAG*; or no Psp proteins present, Δ*pspF*), a clear decrease in Δ*ψ* under pIV stress is observed (Fig. 3) that appears to be ArcB-dependent (Jovanovic *et al*., 2006). As shown in Fig. 3, overexpression of ArcB_WT_, ArcB* or ArcA_WT_ in Δ*pspF*Δ*arcB* or Δ*pspF*Δ*arcA* strains resulted in a clear drop in Δ*ψ* under pIV stress conditions. Interestingly, when we analysed the phosphorelay ArcB and ArcA mutants we no longer observed a drop in Δ*ψ*, suggesting that phosphorylation of ArcA could therefore be a prerequisite for the pIV-dependent drop in pmf.

Notably, in the absence of pIV, overexpression of ArcB* or ArcA_WT_ (which also results in *psp* expression; Fig. 2C) was also sufficient to significantly reduce Δ*ψ* (Fig. 3). In contrast, overexpression of ArcB_WT_, ArcB* phosphorelay mutants (e.g. ArcB*_H717A_) or inactive ArcA (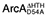) did not reduce Δ*ψ* in the absence of stress (Fig. 3).

### ArcB and PspB interact *in vivo*

To understand how ArcB*-dependent signalling might be integrated into the PspBC-specific signal transduction pathway, we used an adenylate cyclase (Cya)-based bacterial two-hybrid system (BACTH) to study potential Arc–Psp protein–protein interactions *in vivo* (Karimova *et al*., 1998; 2005; and see *Experimental procedures*). We constructed ArcB_WT_, ArcB*, PspA, PspB, PspC and PspF fusion proteins with Cya T18, T18C and T25 fragments (see *Experimental procedures*).

As a control we first analysed the ability of ArcB and ArcB* to self-associate (since ArcB forms dimers; reviewed by Malpica *et al*., 2006) and show that the ArcB_WT_ and ArcB* fusion proteins are capable of self-associating (Table 2). Next, we tested whether ArcB_WT_ or ArcB* could interact with several key members of the Psp regulon (PspA, B, C, F). Additionally we confirmed that known interactions between PspA–PspF, PspA–PspC and PspB–PspC (Table 2 and data not shown) were detected with our fusion proteins (reviewed by Darwin, 2005). We demonstrated that a clear interaction between either ArcB_WT_ or ArcB* (T25-fused protein) occurs with the C-terminal PspB fusion protein (PspB–T18) (Table 2). A Triton X-100 fractionation and Western blotting method was used to demonstrate that the interaction between ArcB, ArcB* and PspB occurs in the IM (using antibodies against Cya T25, Fig. S3Bi or PspB, Fig. S3Bii). As a control, an N-terminal T18C (T18C–PspB) PspB fusion (where T18C is now embedded in the IM) did not detectably interact with ArcB (data not shown). Using an *in vivo* cross-linking assay, we demonstrate that a higher-molecular-weight cross-linked ‘Psp complex’ (labelled cross-linked species in Fig. S3C) can be detected with PspB antibodies in WT and Δ*arcA* strains but not in a Δ*arcB* deletion strain (Fig. S3C), providing further evidence for an interaction between ArcB and PspB.

**Table 2:** Interactions of Psp and ArcB proteins *in vivo*.

	T25 fusions			
T18 fusions	T25–ArcB_WT_	T25–ArcB*	T25–ArcB^LeuZm^	T25–PspC
T18C–ArcB_WT_	+/−	ND	ND	+/−
T18C–ArcB*	ND	+	ND	+/−
PspB–T18	++	++	+	++
PspB^LeuZm^–T18	−	−	−	++
T18C–PspA	+/−	+/−	+/−	+
T18C–PspC	+/−	+/−	+/−	−
T18C–ArcB^LeuZm^	ND	ND	+/−	+/−
T18C–PspF	−	−	−	−

The BACTH system was used to detect the protein–protein interactions between the Psp and ArcB proteins. Negative control: BTH101/pKT25+pUT18C vectors alone (74 ± 4 MU); positive control: BTH101/pKT25-zip+pUT18C-zip (1017 ± 34 MU); interaction estimates including SD were as follows: (+/−) weak interaction (235–350 MU); (+) interaction (350–500 MU); (++) strong interaction (>700 MU); (−) no interaction (≤ 234 MU); ND, not determined. For construction of fusion proteins, growth conditions and mean values with SD, see *Experimental procedures*.

We also found evidence for a weak, but detectable, interaction between ArcB_WT_ or ArcB* and N-terminal T18 fusions to either PspA or PspC (Table 2), but no interaction between ArcB, ArcB* and PspF was detected (Table 2). Since interactions between PspF, PspA, PspB, PspC have previously been established (Adams *et al*., 2003; Elderkin *et al*., 2005; Maxson and Darwin, 2006), we conclude that (potentially regardless of its state) ArcB could interact with members of a Psp[FABC] complex via PspB.

Since both ArcB and PspB contain putative leucine zipper motifs that are most likely positioned at the IM/cytoplasm interface (see Fig. 2A and Fig. S3A), we reasoned that the interaction observed between ArcB and PspB may be occurring directly through these sequences. To determine whether this was indeed the case, we mutated the leucine zipper motifs (LeuZm) of both ArcB (ArcB^LeuZm^; L87A, L90A, L94A, M97A) and PspB (PspB^LeuZm^; L10A L15A L18A) and fused these mutants to T25– and –T18 respectively (see *Experimental procedures* and Table S1). Importantly, the PspB^LeuZm^–T18 fusion protein remained localized within the IM (Fig. S3Bii); however, PspB^LeuZm^ is no longer able to interact with either ArcB_WT_ or ArcB* (Table 2). Interestingly, PspB^LeuZm^ retained the ability to interact with PspC (Table 2), indicating that the interaction with PspC is not dependent on the leucine zipper sequences. These results support a physical interaction between ArcB and PspB mediated by the PspB leucine zipper motif.

Like PspB^LeuZm^–T18, the T25–ArcB^LeuZm^ fusion protein remained localized within the IM (Fig. S4Aii) and importantly retained the ability to activate *pfl* transcription (Fig. S4B), similar to T25–ArcB*. However, T25–ArcB^LeuZm^ showed reduced pIV-independent and pIV-dependent induction of *psp* expression compared with T25–ArcB* (Fig. S4B). Interestingly, the ArcB^LeuZm^ mutant was still able to interact with PspB (albeit not as strongly, Table 2), implying that the site of PspB interaction on ArcB does not solely require the integrity of the putative ArcB leucine zipper motif. Like ArcB_WT_, ArcB^LeuZm^ self-associates (Table 2), suggesting that the ArcB leucine zipper motif does not play a major role in self-association. Finally, ArcB^LeuZm^ retained a very weak interaction with PspA and PspC (Table 2), at a similar level to those observed with ArcB_WT_ or ArcB*, suggesting no role for the ArcB leucine zipper in these interactions.

### ArcB^LeuZm^ is in an active kinase form

**Fig. 4:**
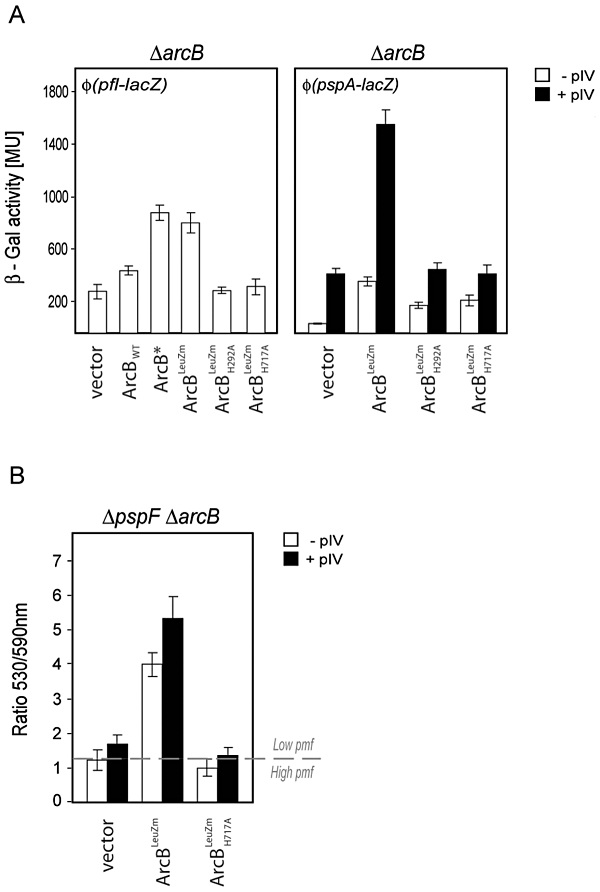
Activities of the ArcB^LeuZm^ protein. A. ArcB LeuZm has an ‘on’ kinase activity and supports induction of *psp* expression. Activities of overexpressed ArcB^LeuZm^ and its corresponding phosphorelay mutants were assessed in a microaerobically grown Δ*arcB* strain (MVA59) also carrying the *pfl–lacZ* (pGJ46) transcriptional fusion construct (left panel). Activities of ArcB^LeuZm^ and its variants were compared with ArcB_WT_ and ArcB*. pIV-independent and -dependent induction of *psp* expression after complementation of the Δ*arcB* mutation (MVA63) with ArcB^LeuZm^ and its variants is presented in the right panel. *pfl* or *psp* expression was measured using β-Gal assays. Vector control was pCA24N. B. ArcB^LeuZm^ reduces cells' Δ*ψ*. *E. coli*Δ*pspF*Δ*arcB* cells (MVA61) expressing ArcB^LeuZm^ and its variants in the presence and absence of pIV were treated with JC-1 dye and changes in Δ*ψ* calculated. An increase in the 530/590 ratio indicates a decrease in Δ*ψ* compared with Δ*pspF*Δ*arcB* strain with the vector plasmid pCA24N.

Since the primary role of the putative LeuZm in ArcB is not in binding to PspB, we decided to investigate the role of this sequence. Initially, we tested the activity of ArcB^LeuZm^ in a Δ*arcB* strain and found that, in contrast to ArcB_WT_, ArcB^LeuZm^ is able to significantly upregulate the *pfl* promoter (Fig. 4A) as does ArcB* (see also Fig. S2B). Importantly, upregulation of the *pfl* promoter by ArcB^LeuZm^ is dependent on the phosphorelay residues H292 and H717; the 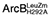 and 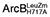 (see *Experimental procedures*, Table S1) do not show elevated *pfl–lacZ* activities (Fig. 4A), but are expressed at similar levels (Fig. S4Ai and data not shown). Overexpression of ArcB^LeuZm^ in a Δ*arcB* strain resulted in elevated *psp* expression in the absence of pIV and full induction in the presence of pIV (Fig. 4A), similar to ArcB* (Fig. 2C). Consistent with the *pfl–lacZ* findings, phosphorelay in the ArcB^LeuZm^ (Fig. 4A, compare ArcB^LeuZm^ with 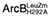 and 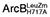) appears critically important for pIV-dependent induction of *psp* expression, but less so for pIV-independent elevation of *psp* expression. Overexpression of ArcB^LeuZm^ in a Δ*pspF*Δ*arcB* strain (Fig. 4B) complemented the *arcB* mutation and reduced Δ*ψ* in the presence and absence of pIV (similar to ArcB*). However as shown in Fig. 4B, overexpression of 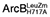 neither complemented the *arcB* mutation in the presence of pIV, nor reduced Δ*ψ* in the absence of pIV, consistent with the inability of 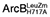 to upregulate *pfl* transcription or stimulates *psp* expression (Fig. 4A).

It seems that ArcB^LeuZm^ resembles ArcB* (an active ArcB form) for the upregulation of *pfl* transcription (compare Fig. S2B and Fig. 4A) and induction of *psp* expression (compare Figs 2C and 4A), as well as its effect on Δ*ψ* (compare Figs 3 and 4B). Introduction of the H292A or H717A mutations in ArcB^LeuZm^ led to the same reduction in activity seen for ArcB*, demonstrating the importance of an intact phosphorelay system for ArcB^LeuZm^ activity.

### PspBC propagates the signal onwards from the Arc system

Since a specific binding interaction between ArcB and PspB is evident, we next explored the relationship between ArcB and PspB in the context of a PspBC-specific signal transduction pathway resulting in *psp* induction.

**Fig. 5:**
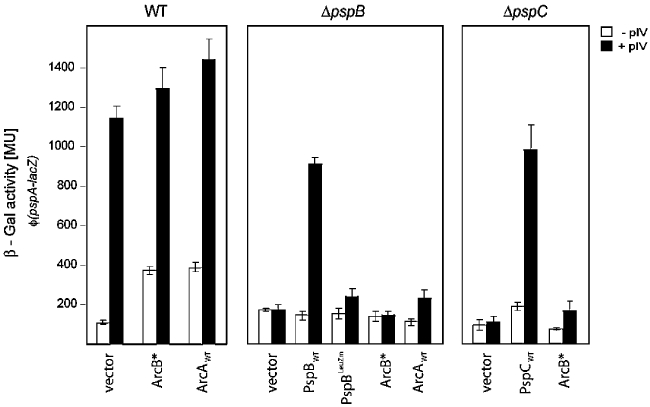
Roles of PspB and PspC in transducing the *psp*-inducing signal(s). *psp* expression was induced by either pIV, ArcB* or ArcA_WT_. Cells were grown under microaerobic conditions and expression of *psp* was measured using β-Gal assays. The Δ*pspB* mutation was complemented with either PspB (pAJM1) or PspB^LeuZm^ (pGJ48), and Δ*pspC* mutation was complemented by PspC (pAJM2). WT – wild-type strain [MC1061 φ(*pspA–lacZ*); MVA4]; vector (WT panel) – pCA24N; vector (Δ*pspB/C* panels) – pBAD18-cam.

In the absence of PspB (Δ*pspB*) neither pIV nor overexpression of ArcB* or ArcA_WT_ significantly induced *psp* expression (Fig. 5). Overexpression of ArcB* in the presence of pIV in Δ*pspB* cells failed to induce *psp* expression (Fig. 5). PspB_WT_ but not PspB^LeuZm^ complemented Δ*pspB* for pIV-dependent induction of *psp* expression (Fig. 5), consistent with the inability of PspB^LeuZm^ to interact with ArcB (Table 2). Importantly, both PspB_WT_ and PspB^LeuZm^ are stably expressed and localized within the IM (Fig. S5). Further, in the absence of PspC (Δ*pspC*), *psp* induction by pIV and by ArcB* was also abolished (Fig. 5). Considering that pIV-dependent induction of *psp* expression involves ArcB and ArcA activities, we suggest that the Arc and Psp systems are integrated via the PspB leucine zipper motif and as such, Arc conditionally forms part of the PspBC-specific signal transduction pathway.

## Discussion

### Conditional requirement for Arc activation in pIV-dependent induction of *psp* expression

In this work, several lines of evidence demonstrate that the ArcAB system in microaerobiosis is required to facilitate pIV-dependent induction of *psp* expression and may potentially contribute to maintaining pmf (via the Psp response). ArcB activation is clearly essential for pIV-dependent induction of *psp* expression as is the integrity of the phosphorelay signalling cascade (which results in phosphorylation of ArcA) and ArcA itself. However, we doubt that the Arc system directly controls expression of the *psp* genes, since neither of the two *E. coli psp* promoters contains recognizable ArcA binding sites and the absence of Arc proteins does not affect transcription of *psp* genes in Δ*pspA* cells (i.e. in the absence of negative control).

In microaerobiosis, Arc-dependent induction of *psp* expression in the presence of pIV correlates with a decrease in Δ*ψ* (pmf), which is dependent both on the phosphorelay activity of ArcB and on ArcA itself. Under pIV stress conditions, the Psp response is not able to conserve pmf when the Arc proteins are absent, therefore we suggest that activation of the ArcAB system helps with generating and/or propagating a pIV-dependent *psp*-inducing signal threshold needed to mount the Psp response. Since the Psp response helps to maintain the cells pmf (and energy status) and is implicated in the growth and virulence of enterobacteria (reviewed by Darwin, 2005; Rowley *et al*., 2006; Darwin, 2007), the cross-talk between the Arc and Psp systems is of physiological relevance given that changes in oxygen availability often prevail during pathogenesis.

The requirement for ArcAB activation in pIV-dependent induction of *psp* expression significantly decreases in aerobiosis and anaerobiosis, consistent with the proposed major importance of the ArcAB regulators in microaerobic growth and in managing transitions between aerobic and anaerobic conditions (Alexeeva *et al*., 2003; Partridge *et al*., 2007). Conditional use of ArcAB can explain results in which involvement of Arc in *psp* induction was not evident under aerobic growth conditions (Seo *et al*., 2007).

We speculate that in anaerobiosis, the more reducing environment in the cytoplasm will decrease the *psp*-inducing signal threshold resulting in increasingly Arc-independent pIV-dependent *psp* expression, consistent with the cellular redox state (as defined by the NADH/NAD ratio) favouring a reducing environment in anaerobiosis versus an oxidizing environment in aerobiosis (de Graef *et al*., 1999). In addition, under anaerobic conditions, the contribution of UQ to the respiratory chain is significantly lower (and is replaced by menaquinone) (Gennis and Stewart, 1996) and so may contribute to a decreased requirement for Arc in pIV-dependent induction of *psp* expression. Hence, Arc-dependent induction of *psp* expression under pIV stress depends on specific growth conditions. Other examples of conditional effects of Arc on certain genes are known (Mika and Hengge, 2005).

### ArcB communicates with PspB

There appear to be two separate actions of the Arc system in pIV-dependent induction of *psp* expression. One is related to activation of ArcA (and subsequent control of ArcA-P target genes) required for full *psp* induction under pIV stress. The other action relates to a more direct effect of activated ArcB that promotes low-level induction of *psp* expression, where ArcB*, in the absence of pIV, can induce low-level *psp* expression in a Δ*arcA* strain as can ArcB* phosphorelay mutants in Δ*arcB* cells. We demonstrated that ArcB interacts with PspB and that this interaction is dependent on the integrity of the PspB leucine zipper motif. Further, mutational analysis revealed that the PspB leucine zipper is important for pIV-dependent induction of *psp* expression, but not required for direct binding interactions between PspB and PspC, suggesting that PspB may be directly involved in receiving (or transducing) an inducing signal from activated ArcB.

The linker region of ArcB connects the *trans*-membrane (TM) domain with the catalytic domain of ArcB (see Fig. 2A and B) and contains a putative leucine zipper motif (conserved leucine residues at positions 73, 80, 87 and 94) and a PAS domain, the former of unknown function for ArcB activity. It has been suggested that Leu87 functions in intramolecular signal propagation in ArcB kinase activation (reviewed by Malpica *et al*., 2006). Here we show that the ArcB leucine zipper motif is functionally important in intramolecular activation of ArcB. Our results indicate that the ArcB leucine zipper mutant (ArcB^LeuZm^) is in a constitutive ‘kinase on’ state, capable of inducing the *pfl* promoter, but not if the phosphorelay residues H292 or H717 are compromised (e.g. 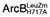). ArcB^LeuZm^ also facilitates pIV-dependent and increases pIV-independent induction of *psp* expression. However, induction of *psp* expression by 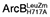 (for example) is significantly lower when compared with ArcB*_H717A_. In addition, the ArcB^LeuZm^ mutant exhibits a decreased affinity for PspB, further suggesting that under specific *psp*-inducing conditions, the leucine zipper motifs of both ArcB and PspB are involved in ArcB–PspB communication.

The PAS domain of ArcB may allow ArcB to respond to a number of factors related to changes in, e.g. oxygen supply and associated metabolic processes. To date only the interaction between ArcB and UQ has been reported (reviewed by Malpica *et al*., 2006). Since ArcB specifically interacts with PspB, in principle this would allow PspB to respond to signals received by ArcB, and vice versa. A similar relationship between signalling and control components has been described for other systems (e.g. for PhoP and PmrD) (Kato *et al*., 2007) where communication often occurs through membrane interactions (Szurmant *et al*., 2008).

### Integration of the Arc system in the PspB/C-specific signalling pathway

*In vivo* it has been shown that PspB, PspC and PspA and PspA and PspF interactions occur under non-stress conditions (reviewed by Darwin, 2005). Here, we show that ArcB and PspB interact, potentially within one or more ArcB–Psp[B/C/A/F] regulatory complexes or subcomplexes. Stress-induced activation of ArcB may result in conformational changes in either PspB or PspC (or both) and presumably through altered protein–protein interactions releases the PspA-imposed negative regulation of PspF.

The precise nature of the signal(s) that induces *psp* expression remains to be determined. It is possible that under stress conditions, besides changes in the integrity of the IM and/or Δ*ψ* (pmf), the redox state of respiratory chain components specifically quinones, may also contribute to Arc-dependent induction of *psp* expression. One possibility is that alongside Arc-independent signalling, *psp*-inducing stimuli reduce UQ and, following an increased diffusion of H^+^ into the cytosol, the consequent dissipation of the pmf, may then activate ArcB. This view is supported by the observation that both UQ and ArcAB are required to detect a drop in Δ*ψ* in the presence of pIV, although UQ may be required for the structural integrity ArcB (Jovanovic *et al*., 2006; this study). Notably induction of *psp* expression and a significant downregulation of aerobic respiration and pmf occur simultaneously in cells undergoing contact-dependent growth inhibition (Aoki *et al*., 2009).

In microaerobiosis, activation of the Arc system greatly facilitates pIV-dependent induction of *psp* expression in a PspBC-dependent manner. However, since part of the induction process is Arc-independent but still PspBC-dependent (Fig. 1) it seems likely that one (or both) of the *psp* encoded sensors (PspB and/or PspC) can receive a signal. Therefore, a ‘double check point’ potentially exists to regulate pIV-dependent induction of *psp* expression. This arrangement of sensors would help the Psp system maintain specificity to stimuli, as described for unorthodox two-component systems which maintain their robustness to noises (Kim and Cho, 2006).

### PspA–PspF is a minimal Psp system

This study clearly shows that in anaerobiosis, pIV-dependent induction of *psp* expression is PspBC-independent, consistent with the observation that some *psp*-inducing stimuli are PspBC-independent (e.g. extreme heat shock, CCCP) or only partially PspBC-dependent (ethanol treatment and hyperosmotic shock) [(reviewed by Model *et al*., 1997; Darwin, 2005); Jovanovic *et al*., 2006]. These results strongly suggest either (i) that PspA (and/or PspF) can directly receive the inducing signal and relieve the PspA-imposed negative regulation or (ii) that other factors substitute for PspBC and ArcAB. That a minimal PspA–PspF system is capable of responding to extracytoplasmic stimulus is consistent with a regulated PspA stress response in Gram-positive bacteria and archebacteria where PspBC are absent [(reviewed by Darwin, 2005); Bidle *et al*., 2008; Vrancken *et al*., 2008]. PspA homologues are found among Gram-positive bacteria, cyanobacteria, archea and higher plants [(reviewed by Darwin, 2005); Bidle *et al*., 2008; Vrancken *et al*., 2008]. In all these organisms, PspA homologues often respond to stress conditions similar to those described for inducing the Psp response (e.g. via extreme heat shock, ethanol treatment, hyperosmotic shock, CCCP) in a partially PspBC-dependent or -independent manner in enterobacteria.

## Experimental procedures

### Bacterial strains and growth conditions

The bacterial strains used in this study are listed in Table S1. Strains were constructed by transduction using the P1_*vir*_ bacteriophage (Miller, 1992) (see Table S1) and were routinely grown in Luria–Bertani (LB) broth or on LB agar plates at 37°C (Miller, 1992). For BACTH assays, strains were grown in LB at 30°C. For *in vivo* cross-linking experiments, strains were grown in minimal medium [50 mM MOPS (pH 7), 2 mM MgSO_4_, 0.5% Glucose (w/v), 10 mM NH_4_Cl, 0.75 mM Na_2_SO_4_, 1.2 mM NH_4_NO_3_, 0.5 mM KH_2_PO_4_] at 30°C. For aerobic growth, overnight cultures of cells were diluted 100-fold into 5 ml of LB in a universal tube with loose fitting caps and shaken at 200 r.p.m. For microaerobic growth, overnight cultures of cells were diluted 100-fold into 5 ml of LB and shaken at 100 r.p.m. For an anaerobic growth, overnight cultures grown at 37°C in a universal tube with tightly closed caps without shaking were transferred (100-fold dilution) into a fully LB-filled suba-sealed universal tube (to avoid any air space) and incubated overnight at 37°C without shaking. The cells were then taken by syringe for β-galactosidase (β-Gal) assays. The pIV secretin was constitutively expressed from pGJ4. For induction of the pBAD *ara* promoter, 0.001% final arabinose (Ara) was added for 1 h. For induction of the *lac* promoter, 0.1 mM isopropyl-β-d-galactopyranoside (IPTG) was added for 1 h. For scoring the *lacZ*^+^ colonies, indicator plates containing 40 µl of 20 mg ml^−1^ stock solution of 5-bromo-4-chloro-3-indolyl-β-d-galactopyranoside (X-gal) and 0.5 mM IPTG were used. Antibiotics were routinely used at the following concentrations: ampicillin (Amp; 100 µg ml^−1^), kanamycin (Kan; 25 µg ml^−1^), chloramphenicol (Cam; 30 µg ml^−1^) and tetracycline (Tet; 10 µg ml^−1^).

### Construction of the *ΔarcB59* mutant

To eliminate the Kan cassette from a Δ*arcB*::Kan mutant (strain MVA59) and construct the marker-less *arcB59* variant (strain MVA92), we used plasmid pCP20 and the method described by Cherepanov and Wackernagel (1995). The resulting strain MVA92 had lost the FRT-flanked Kan resistance gene and the FLP helper plasmid (pCP20) was used to construct the strains MVA93 and MVA94 (see Table S1). The PCR and ParcB/elbB pair of primers were used to verify that *arcB59* mutant in MVA92–94 strains had the correct structure (see Fig. S2D). PCR reactions were carried out either with isolated chromosomal DNA (Qiagen, DNeasy) or with a freshly grown colony suspended in 15 µl of water.

### Measurement of oxygen consumption in bacterial cultures

Oxygen content in bacterial cultures was measured using a non-invasive OxySense 4000B system (Air Monitors). Bacterial cultures grown either in aerobiosis, microaerobiosis or anaerobiosis (see below) were prepared as described above, except that the tubes for dilution of overnight cultures contained an O_2_xyDot sensor (OxySense, Dallas, TX, USA) to detect O_2_ concentrations. The output fluorescence was measured by an OxySense Reader Pen (through the growth tube) and analysed using the OxySense 4000B system expressed either as a percentage or p.p.b. (1000 p.p.b. = 1 mg of O_2_ l^−1^). For oxygen consumption experiments, autocalibrated O_2_xyDot sensors were used and control measurements of ambient air (20.446 ± 1.005%) and the LB medium under either aerobic (8.845 ± 0.965%), microaerobic (4.901 ± 0.181%; air above the medium 17.147 ± 0.624%) or anaerobic conditions (0.075 ± 0.010%) were taken. An optimal signal-to-noise ratio was achieved by measuring in reduced ambient light conditions. Mean values of the oxygen consumption were calculated from 10 measurements with 100 signals captured per sample taken from technical duplicates of three independently grown cultures of each strain.

### DNA manipulations

Plasmids used in this study are shown in Table S1. Using PCR-based site-specific mutagenesis (Quickchange mutagenesis kit, Stratagene) of the plasmid templates pJW5536(−) (ArcB), pJW4364(−) (ArcA) and pAJM1, we constructed plasmids pGJ21-23/27/29-31/33, pGJ45 and pGJ48 respectively (see Table S1). The positions of substituted residues in ArcB and ArcA and the mutant proteins activities are shown in Fig. 2A and Table 1. Plasmid pGJ46 that carries the φ(*pfl–lacZ*) transcription fusion was constructed as shown in Table S1. A DNA fragment carrying the *pfl* promoter region was chosen according to Drapal and Sawers (1995). All constructs were verified by DNA sequencing.

For the BACTH system experiments, the ArcB, ArcB* or ArcB^LeuZm^ proteins were fused to either T25 (plasmid pKT25), T18C (pUT18C) or T18 (pUT18) Cya domains. The corresponding genes were amplified from plasmids pJW5536(−), pGJ23 and pGJ22 using primers that introduce either XbaI–KpnI, HindIII–KpnI or XbaI–EcoRI restriction sites, cloned in pGEM-T Easy (Promega), and then subcloned in frame into multiple cloning sites (MCS) of pKT25, pUT18 and pUT18C respectively. The genes for PspA, PspB, PspC and PspF were amplified by PCR using primers that introduce XbaI–KpnI restriction sites, cloned in pGEM-T Easy and then subcloned in frame into MCS of pKT25, pUT18 and pUT18C. The PspB^LeuZm^ mutant was amplified from plasmid pGJ48 using primers that introduce HindIII–KpnI restriction sites, cloned in pGEM-T Easy and then subcloned in frame into MCS of pUT18. All constructs were verified by DNA sequencing. The production of fusion proteins, following induction by 0.5 mM IPTG at 30°C, was verified using Western blots (see below) with antibodies corresponding to either the fused proteins or Cya T25 (for pKT25 constructs). Transformation of bacteria was performed as described by Miller (1992).

### *In vivo* BACTH

The Cya-based BACTH allows detection of protein–protein interactions *in vivo* and is particularly appropriate for studying interactions among membrane proteins (Karimova *et al*., 1998; 2005). Proteins of interest are fused to the T18 and T25 fragments (which do not function when physically separated) of the catalytic domain of Cya, and co-transformed into the host strain BTH101 (*cya*^-^). Upon interactions of fused proteins, cAMP synthesis is restored leading to transcriptional activation of CAP/cAMP-dependent genes, such as the *lac* operon on the chromosome of the host reporter strain.

The topologies of ArcB (Fig. 2A) (reviewed by Malpica *et al*., 2006), PspA, PspB and PspC (Fig. S3A) (reviewed by Darwin, 2005) were taken into account for construction of the fusion proteins and analysis of results (e.g. a Cya domain fused to the PspB region embedded in IM could interfere with localization of PspB). As a negative control we used pKT25 and pUT18C vectors in the absence of fusion proteins, as a positive control we used pKT25-zip and pUT18C-zip plasmids carrying fused GCN4 leucine-zipper sequence (Karimova *et al*., 1998). After co-transformation of the BTH101 strain with the two plasmids expressing the fusion proteins, selection plates (Kan, Amp, X-gal and IPTG) were incubated at room temperature for 72 h. The levels of the interactions were quantified by β-Gal activity in liquid cultures (see below). LacZ expression threefold above the negative control value was considered a positive interaction signal. For this measurement bacteria were grown in LB medium containing 100 µg ml^−1^ Amp and 50 µg ml^−1^ Kan at 30°C for 16 h, then cultures were diluted 1:25 and grown until the OD_600_ reached ∼0.3, then 0.5 mM IPTG was added and the cells incubated for a further 1 h at 30°C.

### β-Gal assays

Activity from a single-copy chromosomal φ(*pspA–lacZ*) transcriptional fusion was assayed to gauge the level of *psp* expression. The φ(*pspA–lacZ*) transcriptional reporter fusion was introduced as a single copy into the chromosomal *att* site to retain the native *pspA* locus and permit the WT *psp* response. Activity from the plasmid-derived φ(*pfl–lacZ*) transcriptional fusion (pGJ46) was assayed to gauge activity of ArcA-P. Cells were grown overnight at 37°C in LB broth containing the appropriate antibiotic and then diluted 100-fold (initial OD_600_ ∼ 0.025) into the same medium (5 ml). Following incubation to OD_600_ ∼ 0.2–0.3, cultures were induced with Ara or IPTG for 1 h (where appropriate), and then assayed for β-Gal activity as described by Miller (1992). For anaerobically grown cells, β-Gal activity was measured following overnight growth at 37°C with no shaking. For all β-Gal assays, mean values of six independent assays taken from technical duplicates of three independently grown cultures of each strain were used to calculate activity. The data are shown as a mean values with SD error bars.

### Bacterial cell fractionation

The bacterial cultures (5 ml) were separated into soluble and membrane fractions by a lysozyme-EDTA-osmotic shock protocol and IM and OM were selectively extracted with Triton X-100 (Russel and Kaźmierczak, 1993). All the samples were air-dried before adding 30 µl of 4% SDS and 30 µl of Laemlli buffer (Sigma). Samples were analysed by Western blotting (see below).

### Western blot analysis

For the Western blot analysis, bacterial cells were collected (1 ml) at OD_600_ ∼ 0.5. Proteins were separated on either 10%, 12.5% or 15% (SDS)-PAGE and transferred onto PVDF membranes using a semi-dry transblot system (Bio-Rad). Western blotting was performed as described (Elderkin *et al*., 2002) using antibodies to PspF (1:4000 with anti-mouse; Ammersham), PspA (1:10 000 with anti-mouse) (Jones *et al*., 2003), PspB (a gift from P. Model) (1:5000 with anti-rabbit; Ammersham), PspC (a gift from P. Model) (1:5000 with anti-rabbit), pIV (a gift from M. Russel) (1:10 000 with anti-rabbit), ArcB (a gift from D. Georgellis) (1:10 000 with anti-rabbit) and Cya T25 (a gift from D. Ladant) (1:5000 with anti-rabbit). The proteins were detected using ECL plus Western Blotting Detection Kit according to manufacturer's guidelines (GE Healthcare). Images were captured in a FujiFilm – intelligent Dark Box by an image analyser with a charge-coupled device camera (LAS-3000). Densitometry analysis was performed with MultiGauge 3.0 software (FujiFilm USA, Valhalla, NY, USA) and quantification (results expressed in arbitrary units) performed using the AIDA software.

### *In vivo* cross-linking

*In vivo* cross-linking was performed as described (Adams *et al*., 2003) using the thiol-reactive cross-linking reagent dithiobis (succinimydylpropionate) DSP (PIERCE). Cells were grown in minimal medium at 30°C to an OD_600_ ∼ 1.0, harvested and washed in 0.9% NaCl. Proteins were exposed to the cross-linker [100 µM DSP in 125 mM HEPES (pH 7.3)] for 30 min at 25°C. To quench the reaction, 50 µl of 1 M Tris-HCl (pH 8.0) was added to the reaction mix and the samples then transferred to ice for 5 min. Proteins were separated on a 7.5% SDS-PAGE (run at 200 V for 50 min) and the cross-linked species detected using antibodies specific to PspB (α-PspB) as described above.

### Confocal fluorescence microscopy to assess membrane electron potential (Δ*ψ*)

The Δ*ψ* component of pmf was measured as described by Becker *et al*. (2005), with the following modifications. Cells from an overnight LB culture were subcultured into fresh LB to an initial OD_600_ ∼ 0.025 and grown to an OD_600_ of 0.8. Bacterial culture (1 ml) was centrifuged and then re-suspended in 1 ml of permeabilization buffer (10 mM Tris pH 7.5, 1 mM EDTA, 10 mM glucose). Then 2 µl of 5 mg ml^−1^ 1,1′,3,3′-tetraethylbenzimidazolyl-carbocyanine iodine (JC-1) (Molecular Probes) was added and the preparation incubated for 30 min at room temperature. Cells were then centrifuged and re-suspended in 500 µl of permeabilization buffer. Microscope slides were prepared as follows: 80 µl of molten 0.8% agarose was pipetted onto a clean microscope slide, covered with a coverglass pre-treated with sigmacote (Sigma) and left to dry at room temperature for 30 min, prior to removing the coverglass. To obtain a thin layer of agarose, the microscope slide was left to dry overnight at 37°C. Fluorescent bacteria were examined using a Leica TCS-NT confocal microscope (Leica Microsystems, Germany) equipped with a krypton/argon laser with an excitation wavelength of 485 nm. Leica confocal software (Leica Microsystems, Germany) was used to calculate the green/red fluorescence emission ratio from 100 individual cells taken from technical duplicates of three independently grown cultures of each strain. The threshold to distinguish low pmf from high pmf was chosen based on the Δ*ψ* (pmf under given experimental conditions) of the WT strain under normal (non-stress) growth conditions.
