# Supplementary material for: Physical, functional and conditional interactions between ArcAB and phage shock proteins upon secretin-induced stress in Escherichia coli
Source: Mol Microbiol. 2009 Aug 4;74(1):16–28. doi: 10.1111/j.1365-2958.2009.06809.x (PMC2764110; doi:10.1111/j.1365-2958.2009.06809.x)
Supplement: Supplementary file 1 [file mmi0074-0016-SD1.pdf]

Supporting informations

Tables

Table S1. *E. coli* K-12 strains and plasmids used in this study

Strain or plasmid	Relevant characteristics	Reference
**Strain**		
MG1655	Wild type	CGSC# 7740
MG1655 Δ*pspF*	MG1655 Δ*pspF*	Lloyd *et al.*, 2004
MVA42	MG1655 Δ*pspA* Δ*pspG*::Kan (kan^r)	Lloyd *et al.*, 2004
MVA59	MG1655 Δ*arcB*::Kan (kan^r)	Jovanovic *et al.*, 2006
JWK4364	BW25113 Δ*arcA*::Kan (kan^r)	Baba *et al.*, 2006
MVA70	MG1655 Δ*arcA*::Kan (kan^r)	This work MG1655×JWK4364
MVA61	MG1655Δ*pspF* Δ*arcB*::Kan (kan^r)	Jovanovic *et al.*, 2006
MVA80	MG1655Δ*pspF* Δ*arcA*::Kan (kan^r)	This work MG1655×JWK4364
MVA44	MG1655 ϕ(*pspA-lacZ*) (amp^r)	Jovanovic *et al.*, 2006
MVA45	MG1655 Δ*pspBC* ϕ(*pspA-lacZ*) (amp^r)	Jovanovic *et al.*, 2006
MVA63	MVA44 Δ*arcB*::Kan (amp^r, kan^r)	Jovanovic *et al.*, 2006
MVA79	MVA44 Δ*arcA*::Kan (amp^r, kan^r)	This work MVA44×JWK4364
MVA27	MG1655Δ*pspA* ϕ(*pspA-lacZ*) (amp^r)	Jovanovic *et al.*, 2006
MVA62	MVA27 Δ*arcB*::Kan (amp^r, kan^r)	Jovanovic *et al.*, 2006
MVA84	MVA27 Δ*arcA*::Kan (amp^r, kan^r)	This work MVA27×JWK4364
JWK5536	BW25113 Δ*arcB*::Kan (kan^r)	Baba *et al.*, 2006
MVA83	MVA45 Δ*arcB*::Kan (amp^r, kan^r)	This work MVA45×JWK5536
MVA92	MVA59 Δ*arcB59* (kan^s, amp^s, cam^s)	This work MVA59×pCP20
MVA93	MVA92 ϕ(*pspA-lacZ*) (amp^r)	This work MVA92×MVA44
MVA94	MVA93 Δ*arcA*::Kan (amp^r, kan^r)	This work MVA93× JWK4364
MVA4	MC1061 ϕ(*pspA-lacZ*) (amp^r)	Engl *et al.*, 2009
L65	K561 Δ*pspB*::Kan (kan^r)	A gift from P. Model
J136	K561 Δ*pspC*::Kan (kan^r)	A gift from P. Model
MVA12	MVA4 Δ*pspB*::Kan (amp^r, kan^r)	This work MVA4×L65
MVA13	MVA4 Δ*pspC*::Kan (amp^r, kan^r)	This work MVA4×J136
MVA77	MVA4 Δ*arcB*::Kan (amp^r, kan^r)	This work MVA4×JWK5536
JWK1328	BW25113 Δ*fnr*::Kan (kan^r)	Baba *et al.*, 2006
JWK1213	BW25113 Δ*narX*::Kan (kan^r)	Baba *et al.*, 2006
JWK1212	BW25113 Δ*narL*::Kan (kan^r)	Baba *et al.*, 2006
JWK2453	BW25113 Δ*narQ*::Kan (kan^r)	Baba *et al.*, 2006
JWK2181	BW25113 Δ*narP*::Kan (kan^r)	Baba *et al.*, 2006
JWK4023	BW25113 Δ*soxS*::Kan (kan^r)	Baba *et al.*, 2006
JWK4024	BW25113 Δ*soxR*::Kan (kan^r)	Baba *et al.*, 2006
JWK3933	BW25113 Δ*oxyR*::Kan (kan^r)	Baba *et al.*, 2006
JWK0669	BW25113 Δ*fur*::Kan (kan^r)	Baba *et al.*, 2006
EC1	MVA4 Δ*fnr*::Kan (amp^r, kan^r)	This work MVA4×JWK1328
EC2	MVA4 Δ*narX*::Kan (amp^r, kan^r)	This work MVA4×JWK1213
EC3	MVA4 Δ*narL*::Kan (amp^r, kan^r)	This work MVA4×JWK1212
EC4	MVA4 Δ*narQ*::Kan (amp^r, kan^r)	This work MVA4×JWK2453
EC5	MVA4 Δ*narP*::Kan (amp^r, kan^r)	This work MVA4×JWK2181
EC6	MVA4 Δ*soxS*::Kan (amp^r, kan^r)	This work MVA4×JWK4023
EC7	MVA4 Δ*soxR*::Kan (amp^r, kan^r)	This work MVA4×JWK4024
EC8	MVA4 Δ*oxyR*::Kan (amp^r, kan^r)	This work MVA4×JWK3933
EC9	MVA4 Δ*fur*::Kan (amp^r, kan^r)	This work MVA4×JWK0669
BTH101	*cya*$^-$, *lac*$^+$	A gift from D. Ladant
XL1-Blue	tet^r	Laboratory collection

**Plasmid**		
pBR325D	Recombinant cloning vector (cam^r, tet^r, amp^r)	Laboratory collection
pGJ4	P_{lacUV5}-*gIV* (pIV) (tet^r)	Jovanovic *et al.*, 2006
pCA24N	Expression vector, $P_{T5/lac}$ promoter (cam^r)	Kitagawa *et al.*, 2005
pJW5536(-)	$P_{T5/lac}$-*6xhis-arcB* (ArcB wild type), *lacI*^{q} (cam^r)	Kitagawa *et al.*, 2005
pGJ21	pJW5536(-) encodes $ArcB_{H717A}$ (cam^r)	This work
pGJ22	pJW5536(-) encodes $ArcB^{LeuZm}$ (L87A L90A L94A M97A) (cam^r)	This work
pGJ23	pJW5536(-) encodes $ArcB_{C180A/C241A}$ (ArcB*) (cam^r)	This work
pGJ27	pJW5536(-) encodes $ArcB*_{H717A}$ (cam^r)	This work
pGJ29	pJW5536(-) encodes $ArcB^{LeuZm}{}_{H717A}$ (cam^r)	This work
pGJ30	pJW5536(-) encodes $ArcB*_{H292A}$ (cam^r)	This work
pGJ31	pJW5536(-) encodes $ArcB^{LeuZm}{}_{H292A}$ (cam^r)	This work
pGJ33	pJW5536(-) encodes $ArcB_{H292A}$ (cam^r)	This work
pJW4364(-)	$P_{T5/lac}$-*6xhis-arcA* (ArcA wild type), *lacI*^{q} (cam^r)	Kitagawa *et al.*, 2005
pGJ45	pJW4364(-) encodes $ArcA_{D54A/\Delta HTH}$ (cam^r)	This work
pRS415	Promoter-less *lac* transcription fusion vector (amp^r)	Simons *et al.*, 1987
pGJ46	490 bp of *pfl* promoter region amplified by PCR from MG1655 chromosome and cloned into the vector pRS415 (*Eco*RI-*Bam*HI) creating ϕ(*pfl-lacZ*) (amp^r)	This work
pBAD18-cam	Expression vector, pBAD *ara* promoter (cam^r)	A gift from J. Beckwith
pAJM1	pBAD18-cam with *pspB* cloned into MCS (cam^r)	A gift from A. Mayhew
pGJ48	pAJM1 encodes $PspB^{LeuZm}$ (L10A L15A L18) (cam^r)	This work
pAJM2	pBAD18-cam with *pspC* cloned into MCS (cam^r)	A gift from A. Mayhew
pKT25	IPTG-inducible vector containing the T25 domain of Cya upstream of the MCS (kan^r)	A gift from D. Ladant
pUT18C	IPTG-inducible vector containing the T18 domain of Cya upstream of the MCS (amp^r)	A gift from D. Ladant
pUT18	IPTG-inducible vector containing the T18 domain of Cya downstream of the MCS (amp^r)	A gift from D. Ladant
pKT25-zip	GCN4 leucine zipper fusion to the C-terminus of the T25 domain of Cya in pKT25 (kan^r)	A gift from D. Ladant
pUT18C-zip	GCN4 leucine zipper fusion to the C-terminus of the T18 domain of Cya in pUT18C (amp^r)	A gift from D. Ladant
pCP20	*FLP*$^+$, λ *c*I857$^+$, λ p_R Rep^{ts}, (amp^r, cam^r)	Cherepanov and Wackernagel, 1995
pGEM-T Easy	Cloning vector (amp^r)	Promega

Figures

Fig. S1

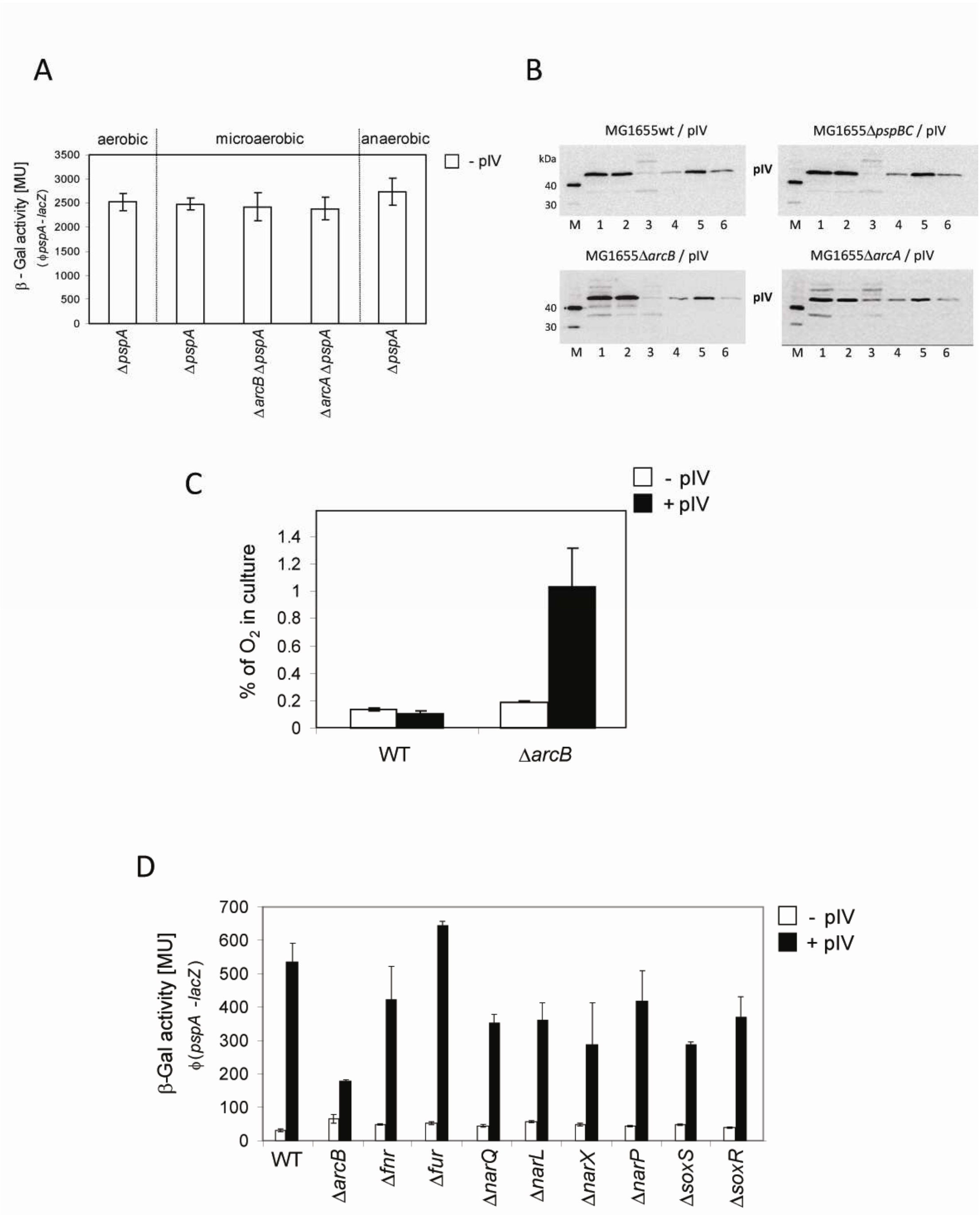

Fig. S2

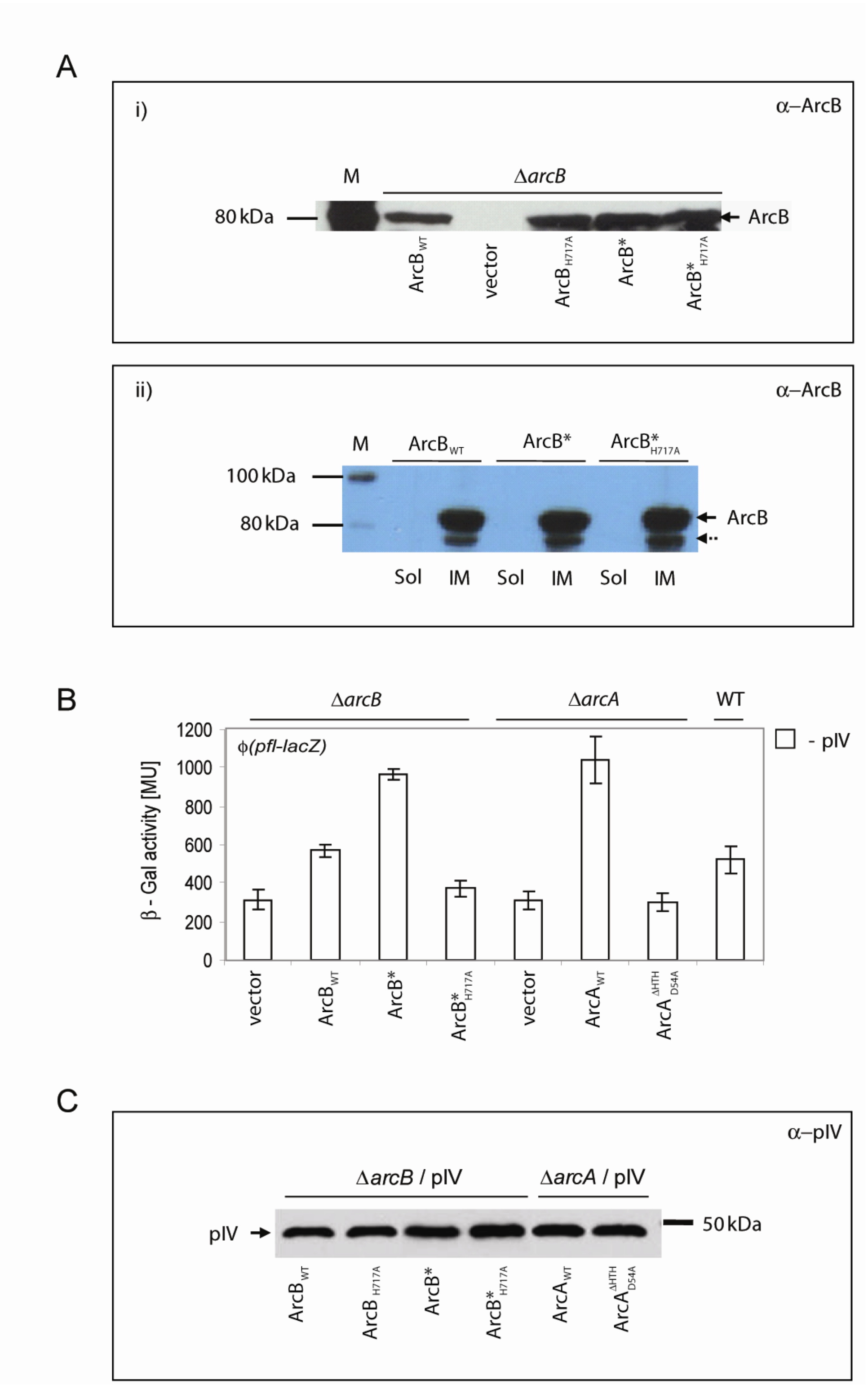
A
i)
α–ArcB
M
ΔarcB
80 kDa
ArcB
ArcB WT
vector
ArcB H717A
ArcB*
ArcB* H717A
ii)
α–ArcB
M
ArcB WT
ArcB*
ArcB* H717A
100 kDa
80 kDa
ArcB
Sol
IM
Sol
IM
Sol
IM
B
ΔarcB
ΔarcA
WT
- pIV
ϕ(pfl-lacZ)
β - Gal activity [MU]
1200
1000
800
600
400
200
0
vector
ArcB WT
ArcB*
ArcB* H717A
vector
ArcA WT
ArcA ΔHTH D54A
C
α–pIV
ΔarcB / pIV
ΔarcA / pIV
pIV
50 kDa
ArcB WT
ArcB H717A
ArcB*
ArcB* H717A
ArcA WT
ArcA ΔHTH D54A

Fig. S2

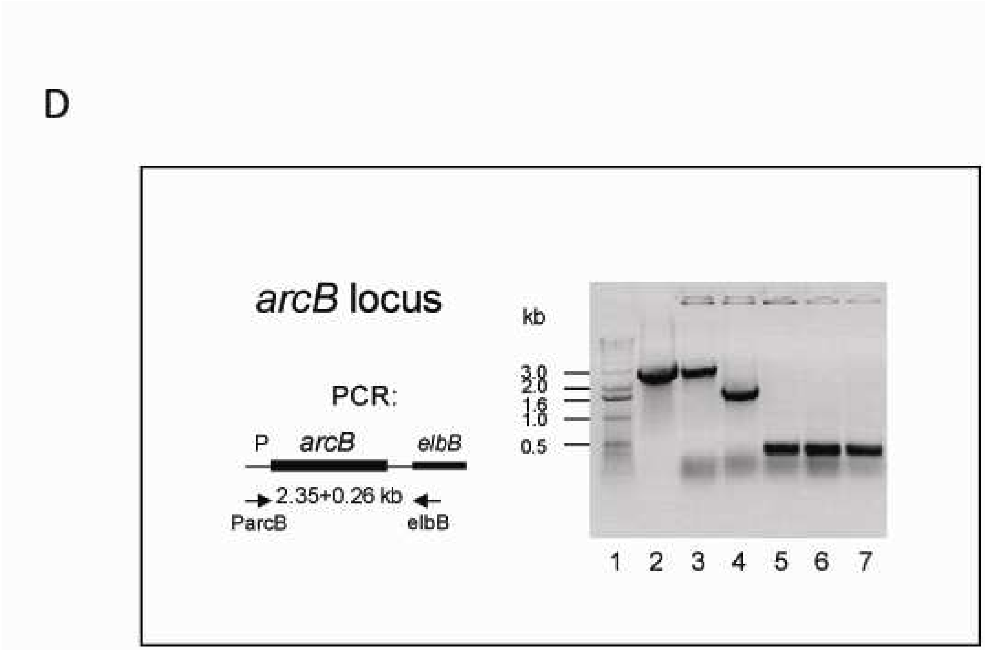
D
arcB locus
PCR:
P
arcB
elbB
2.35+0.26 kb
ParcB
elbB
kb
3.0
2.0
1.6
1.0
0.5
1 2 3 4 5 6 7

Fig. S3

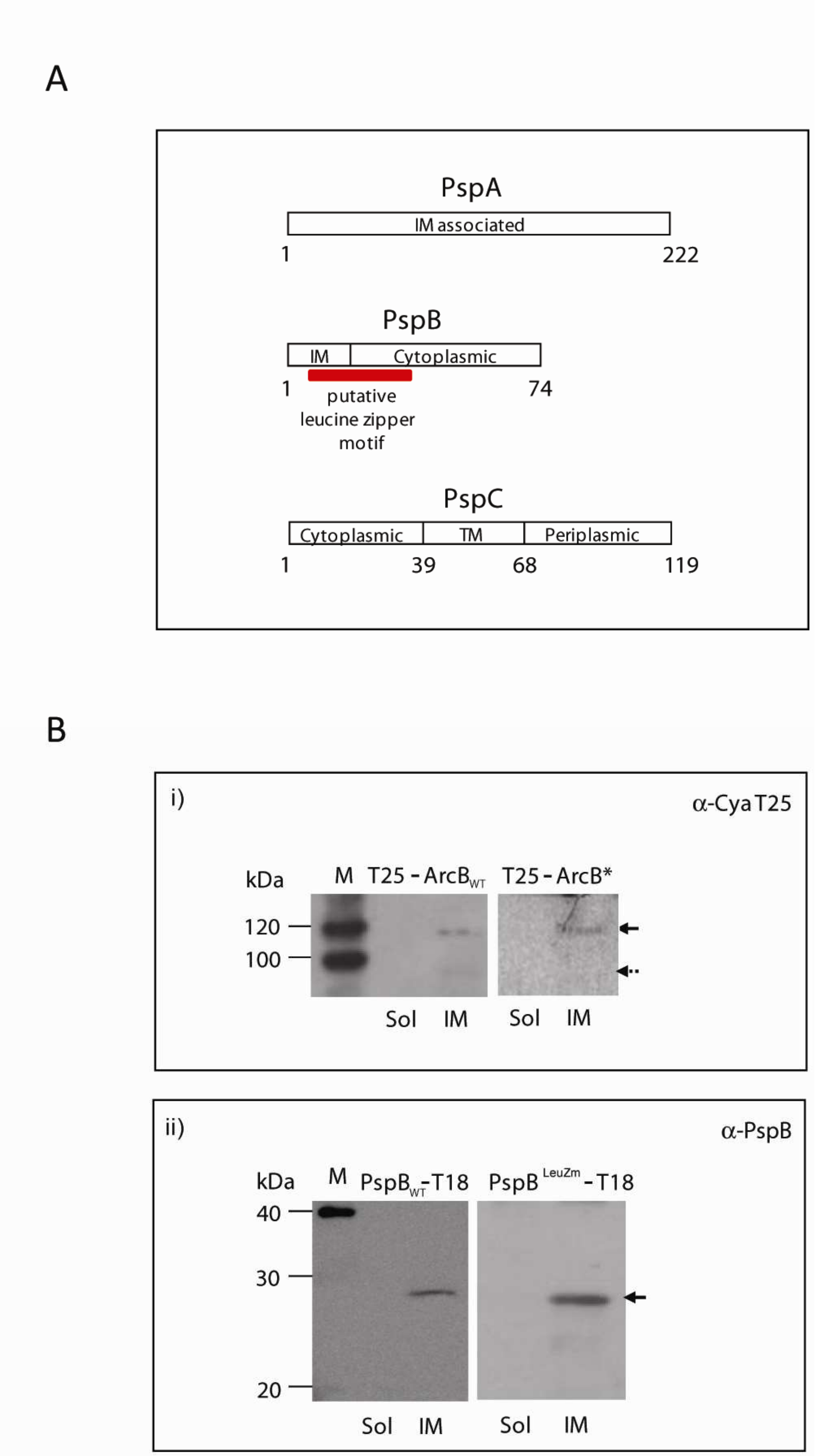
A
PspA
IM associated
1
222
PspB
IM
Cytoplasmic
1
74
putative
leucine zipper
motif
PspC
Cytoplasmic
TM
Periplasmic
1
39
68
119
B
i)
α-Cya T25
kDa
M
T25 - ArcB
T25 - ArcB*
120
100
Sol
IM
Sol
IM
ii)
α-PspB
kDa
M
PspB-T18
PspB LeuZm - T18
40
30
20
Sol
IM
Sol
IM

Fig. S3

C

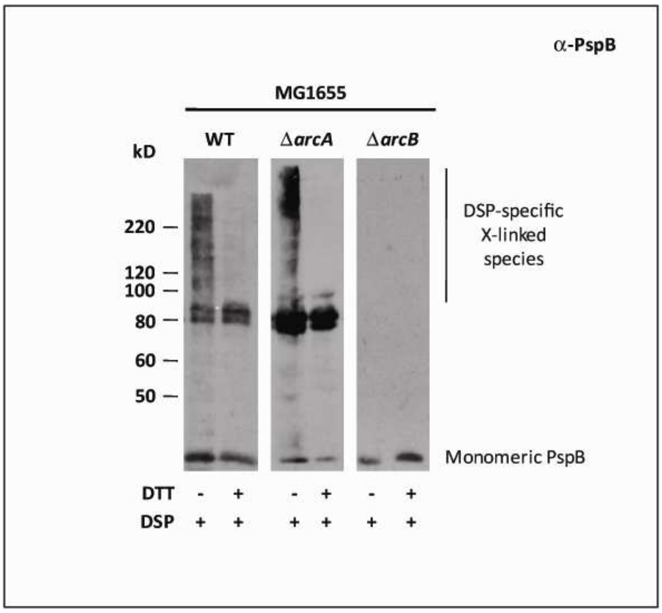
α-PspB
MG1655
WT
ΔarcA
ΔarcB
kD
220
120
100
80
60
50
DSP-specific
X-linked
species
Monomeric PspB
DTT - + - + - +
DSP + + + + + +

Fig. S4

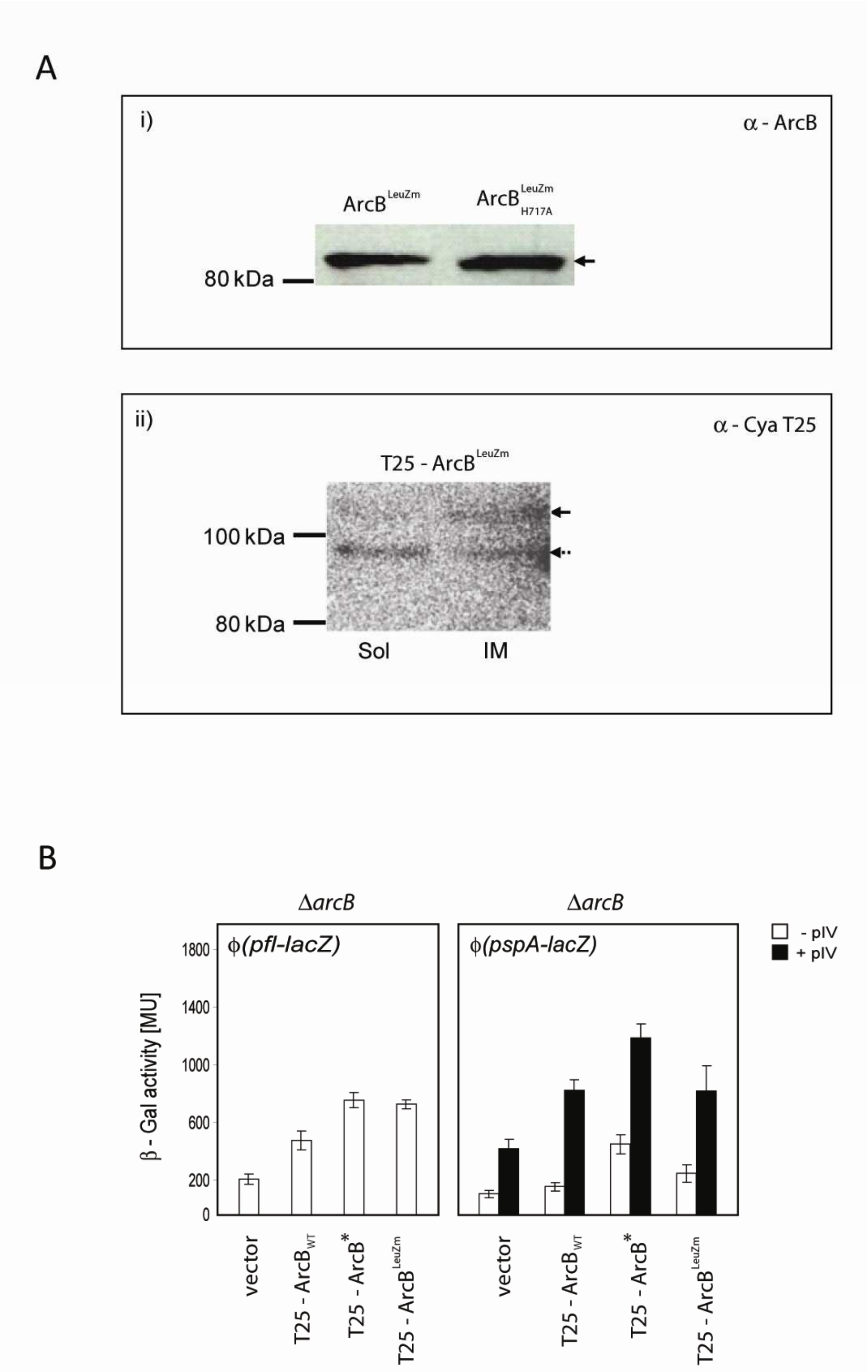
A
i)
α - ArcB
ArcB LeuZm
ArcB LeuZm H717A
80 kDa
ii)
α - Cya T25
T25 - ArcB LeuZm
100 kDa
80 kDa
Sol
IM
B
ΔarcB
ΔarcB
ϕ(pfl-lacZ)
ϕ(pspA-lacZ)
- pIV
+ pIV
β - Gal activity [MU]
1800
1400
1000
600
200
0
vector
T25 - ArcB WT
T25 - ArcB*
T25 - ArcB LeuZm

Fig. S5

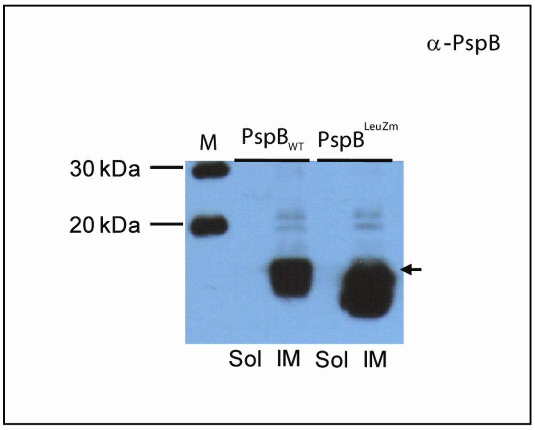

Figure legends

Fig. S1. A) *psp* expression in Δ*pspA* strains grown under different conditions. *psp* expression in *E. coli* Δ*pspA* cells [MG1655 Δ*pspA* ϕ(*pspA-lacZ*); MVA27] grown under aerobic, microaerobic or anaerobic conditions and in Δ*pspA* Δ*arcB* and Δ*pspA* Δ*arcA* cells as measured using a β-Gal assay (see Experimental procedures). **B)** pIV production under microaerobic conditions. pIV expression and its subcellular localisation are not affected in Δ*pspBC*, Δ*arcB* or Δ*arcA* mutants compared to WT. The bacterial cells expressing pIV from pGJ4 were fractionated using a Triton X-100-based extraction method (see Experimental procedures) and analysed by Western blotting using antibodies specific to pIV (α-pIV). M, MW marker, 1, Whole cell, 2, Membranes (IM+OM), 3, Soluble fraction, 4, IM fraction, 5, OM fraction, 6, Insoluble fraction (protein aggregates). The molecular weight of pIV is 44.6 kDa (arrow). **C)** Production of pIV causes different oxygen consumption of ArcB mutant in microaerobiosis. Oxygen consumption is measured using O_2xyDot sensor as described in Experimental procedures. The cultures were grown from an initial OD_{600}~0.025 for 2.5 hours to OD_{600}~0.6 with a similar growth rates (final ODs after 2.5 hrs were WT, 0.624; WT/pIV, 0.706; Δ*arcB*, 0.611; Δ*arcB*/pIV, 0.523) and results were estimated relative to WT growth rate. Percentage of oxygen in bacterial cultures is presented, and calculated in mg/lit were as follows: WT (0.065±0.004), WT/pIV (0.056±0.002), Δ*arcB* (0.095±0.009), Δ*arcB*/pIV (0.418±0.014). WT – wild type strain (MG1655). **D)** Effect of global regulator mutants on pIV-dependent induction of *psp* expression. *psp* expression in different mutants (background strain MVA4) grown microaerobically was measured using β-Gal assay (see Experimental procedures). WT – wild type strain (MVA4).

Fig. S2. A) Expression and localisation of WT ArcB and ArcB mutants. i) ArcB or ArcB mutants overexpressed in a Δ*arcB* strain grown microaerobically (see Fig. 2C) were detected by Western blotting and antibodies against ArcB (α-ArcB). The molecular weight of ArcB is 87.8 kDa (arrow). M – molecular weight marker; vector – pCA24N. Densitometry quantified the expression of ArcB proteins and the relative amounts presented in arbitrary units were as follows: $ArcB_{WT}$, 2.02; $ArcB_{H717A}$, 2.15; ArcB*, 2.46; $ArcB*_{H717A}$, 2.17. The overall variability in ArcB proteins expression is

~1.21-fold. ii) Triton-X100 fractionated bacterial cells overexpressing $ArcB_{WT}$ and ArcB mutants (arrow) were analysed by Western blotting and antibodies against ArcB (α-ArcB) (see Experimental procedures). We suspect that ArcB degradation product was detected as well (hacked arrow), since observed with antibodies against ArcB and Cya T25 (see Fig. S3B i, and S4A ii). M – molecular weight marker; Sol – soluble fraction, IM – IM fraction. **B)** Control of ArcA-P activated *pfl* expression by Arc proteins. Activities of overexpressed Arc proteins were assessed by β-Gal assays in microaerobically grown cells containing the plasmid (pGJ46) borne *pfl-lacZ* transcriptional fusion, ϕ(*pfl-lacZ*) (see Experimental procedures). WT – MG1655; vector – pCA24N. **C)** Production of pIV in cells co-expressing the Arc proteins. pIV production in microaerobically grown cells co-expressing Arc proteins (see Fig. 2C) was detected by Western blotting and antibodies against pIV (α-pIV) (see Experimental procedures). The molecular weight of pIV is 44.6 kDa (arrow). Densitometry quantified the expression of pIV and the relative amounts presented in arbitrary units were as follows: $ArcB_{WT}$, 2.08; $ArcB_{H717A}$, 2.06; ArcB*, 2.22; $ArcB*_{H717A}$, 2.40; $ArcA_{WT}$, 2.18; $ArcA_{D54A}^{\Delta HTH}$, 2.16. The overall variability in pIV expression is ~1.16-fold. **D)** Colony PCR amplification of an *arcB* locus in different backgrounds of MG1655 strain. PCR amplifications of an *arcB* locus was carried out using a pair of primers specific for the *arcB* promoter region (ParcB) and 5' region of an adjacent *elbB* gene, and either isolated chromosomal DNA (lane 2) or the whole DNA from a bacterial night culture (lanes 3-8) as a template (see Experimental procedures). Amplified DNA fragments were run on 0.8% agarose gel electrophoresis. Expected mobilities of DNA fragments were as follows: WT *arcB*, ~2.6 kb (lanes 2-3); Δ*arcB*::Kan, ~1.7 kB (lane 4); Δ*arcB59*, ~0.4 kb (lanes 5-8). Lanes: 1, 1 kb molecular weight marker (Invitrogene); 2-3, MG1655; 4, MVA59; 5, MVA92; 6, MVA93; 7, MVA94.

Fig. S3. Expression and localisation of ArcB and PspB Cya-fusion proteins. A) Schematic representation of PspA, PspB and PspC topologies according to Kleerebezem *et al.*, (1996), Dworkin et al., (2000), and Jones et al., (2003). IM – embedded in an IM, TM – trans-membrane domain. **B)** ArcB and PspB interact in the IM. i) and ii) Western blot analysis of Triton-X100 fractionated BTH101 cells demonstrating that the fusion proteins T25-$ArcB_{WT}$, T25-ArcB*, PspB-T18, and

$PspB^{LeuZm}$-T18 localise within the IM correctly (see Experimental procedures). i) α-Cya T25 was used to detect ArcB fusion proteins (arrow, ~114 kDa) or ii) α-PspB was used to detect the PspB fusion proteins (arrow, ~26 kDa). Sol – soluble fraction; IM – inner membrane fraction; M – molecular weight marker; hacked arrow – possible degradation product of ArcB. **C)** Using *in vivo* cross-linking (as described in *Experimental procedures*) we showed that PspB antibodies recognise a higher molecular weight complex in a MG1655 WT and Δ*arcA* mutant strain but not in a Δ*arcB* strain, providing further evidence that an interaction between ArcB and PspB indeed occurs.

Fig. S4. A) i) Expression of $ArcB^{LeuZm}$ and its phosphorelay mutant variant in Δ*arcB* cells grown microaerobically. Arc proteins were overexpressed and detected (arrow) using Western blotting and antibodies against ArcB (α-ArcB). Densitometry quantified the expression of ArcB proteins and the relative amounts presented in arbitrary units were as follows: $ArcB^{LeuZm}$, 2.12; $ArcB_{H717A}{}^{LeuZm}$, 2.51. The overall variability in ArcB mutants expression is ~1.18-fold. ii) Localisation of the T25-$ArcB^{LeuZm}$. Localisation of the fusion protein in BTH101 was assessed using Triton X-100-based cell fractionation method and Western blot analysis (see Experimental procedures). Antibodies against Cya T25 (α-Cya T25) were used to detect the fusion protein (arrow). Sol – soluble fraction; IM – inner membrane fraction; hacked arrow – possible degradation product of ArcB. **B)** Functionalities of T25-$ArcB_{WT}$, T25-ArcB* and T25-$ArcB^{LeuZm}$ fusion proteins. Activity of overexpressed T25-$ArcB_{WT}$, T25-ArcB* or T25-$ArcB^{LeuZm}$ fusion protein was measured in a microaerobically grown Δ*arcB* strain (MVA92) containing the ArcA-P-regulated *pfl-lacZ* transcriptional fusion plasmid (pGJ46) (left panel). Induction of *psp* expression in Δ*arcB* strain (MVA93) by T25-$ArcB_{WT}$, T25-ArcB* or T25-$ArcB^{LeuZm}$, either in the absence or presence of pIV, is presented in the right panel. For both panels: *pfl* or *psp* expression was measured using β-Gal assay (see Experimental procedures). vector – pCA24N.

Fig. S5. Expression and localisation of PspB and $PspB^{LeuZm}$. The PspB and $PspB^{LeuZm}$ were overexpressed (0.1% Ara) in a Δ*pspB* strain grown microaerobically. The bacterial cells were fractionated by using a Triton-X100-based extraction method, and analysed by Western blotting using antibodies against PspB (α-PspB) (see

Experimental procedures). Molecular weight of PspB is ~8 kDa (arrow). M – MW marker; Sol – soluble fraction, IM – IM fraction.

References:

Baba, T., Ara, T., Hasegawa, M., Takai, Y., Okumura, Y., Baba, M., *et al*. (2006) Construction of *Escherichia coli* K-12 in-frame, single-gene knockout mutants: the Keio collection. *Mol Syst Biol* **2:** 2006.0008.

Cherepanov, P.P., and Wackernagel, W. (1995) Gene disruption in *Escherichia coli*: TcR and KmR cassettes with the option of Flp-catalyzed excision of the antibiotic-resistance determinant. *Gene* **158:** 9–14.

Dworkin, J., Jovanovic, G., and Model, P. (2000) The PspA protein of *Escherichia coli* is a negative regulator of sigma(54)-dependent transcription. *J Bacteriol* **182:** 311–319.

Engl, C., Jovanovic, G., Lloyd, L.J., Murray, H., Ying, L., Errington, J., and Buck, M. (2009) *In vivo* localizations of membrane stress controllers PspAand PspG in *Escherichia coli*. *Mol Microbiol*. **73:** 382–396.

Jones, S.E., Lloyd, L.J., Tan, K.K., and Buck, M. (2003) Secretion defects that activate the phage shock response of *Escherichia coli*. *J Bacteriol* **185:** 6707–6711.

Jovanovic, G., Lloyd, L.J., Stumpf, M.P.H., Mayhew, A.J., and Buck, M. (2006) Induction and function of the phage shock protein extracytoplasmic stress response in *Escherichia coli*. *J Biol Chem* **281:** 21147–21161.

Kleerebezem, M., Crielaard, W., and Tommassen, J. (1996) Involvement of stress protein PspA (phage shock protein A) of *Escherichia coli* in maintenance of the protonmotive force under stress conditions. *EMBO J* **15:** 162–171.

Kitagawa, M., Ara, T., Arifuzzaman, M., Ioka-Nakamichi, T., Inamoto, E., Toyonaga, H., *et al*. (2005) Complete set of ORF clones of *Escherichia coli* ASKA library (a complete set of *E. coli* K-12 ORF archive): unique resources for biological research. *DNA Res* **12:** 291–299.

Lloyd, L.J., Jones, S.E., Jovanovic, G., Gyaneshwar, P., Rolfe, M.D., Thompson, A., *et al*. (2004) Identification of a new member of the phage shock protein response in *Escherichia coli*, the phage shock protein G (PspG). *J Biol Chem* **279:** 55707–55714.

Simons, R.W., Houman, F., and Kleckner, N. (1987) Improved single and multicopy *lac*-based cloning vectors for protein and operon fusions. *Gene* **53:** 85–96.
